# Effects of ultraviolet radiation as a climate variable on the geographic distribution of *Oryza sativa* under climate change based on Biomod2

**DOI:** 10.3389/fpls.2025.1552770

**Published:** 2025-04-16

**Authors:** Rulin Wang, Xiang Guo, Yanling Song, Yuangang Cai, Yuhan Wu, Mingtian Wang

**Affiliations:** ^1^ Water-Saving Agriculture in Southern Hill Area Key Laboratory of Sichuan Province, Chengdu, China; ^2^ Sichuan Provincial Rural Economic Information Center, Chengdu, China; ^3^ Institute of Plateau Meteorology, China Meteorological Administration, Chengdu, China; ^4^ Sichuan Province Agro-meteorological Center, Chengdu, China; ^5^ Chinese Academy of Meteorological Sciences, Beijing, China; ^6^ Mianyang Bureau of Meteorology, Mianyang, China; ^7^ College of Life Science, China West Normal University, Nanchong, China; ^8^ Sichuan Meteorological Observatory, Chengdu, China

**Keywords:** biomod2, climate change, potential geographic distributions, *Oryza sativa*, ensemble model, climate-suitable region

## Abstract

**Introduction:**

*Oryza sativa* is one of the most important cereal crops globally.

**Methods:**

The aim of this study was to map areas suitable for the growth and conservation of *O. sativa* under current and future climatic conditions, and to observe the effects of UV variables on the distribution area of *O. sativa*.

**Results:**

Based on species distribution records, we used the Biomod2 platform to combine climate data, future shared socioeconomic pathways, and elevation data. The ensemble model (EM) was constructed by screening multiple species distribution models (SDMs), including RF, GBM, ANN, and MARS. The ROC value of the joint model is greater than 0.95, indicating that the model has high reliability and accuracy. Mean annual temperature (bio01), temperature seasonality (bio04), minimum temperature in the coldest month (bio06), mean temperature of coldest quarter (bio11), human footprint and human activity impact index (hfv2geo1) and annual average ultraviolet radiation (uvb1annualmeanuv.b) were the most important environmental variables affecting the suitable distribution area of *O. sativa*. Under the current climate conditions, the suitable habitats of *O. sativa* are mainly distributed in the south of the Yangtze River. In the future climate scenario, the total suitable habitat area of *O. sativa* tended to decrease, but the suitable distribution area under the influence of UV was larger than that without UV.

**Discussion:**

Climate change will significantly affect the potential distribution of *O. sativa* in China and increase its extinction risk. Therefore, it is necessary to provide a reference for the conservation, management, introduction and cultivation of food crops in China.

## 1 Introduction

Climate change is a persistent and escalating process, undeniably becoming one of the main drivers of changes in species distribution and biodiversity (Pigot et al 2023). It has a profound impact on species’ structure, function, and ecological traits (Sintayehu 2018). This impact has become a major focus in global research on species spatial patterns, particularly in terms of plant distribution. The response of vegetation to climate change and the resulting shifts in plant distribution areas are important areas of study (Chen et al. 2022; O’Connor et al 2020). Climate factors are crucial in determining plant distribution ranges, which can shift with changing climates. The IPCC (Intergovernmental Panel on Climate Change) notes that climate change will affect most natural systems with varying degrees of intensity, threatening food production and increasing the risk of natural disasters (Arias et al 2021). Research indicates that future climate change will alter the suitable climatic regions for plants, posing a serious threat to biodiversity (Tian and Jiang 2015).

Most studies on changes in plant species distribution and their future trends primarily focus on climate factors, with less attention given to the synergistic effects of habitat and climate change (Di Febbraro et al. 2023; Vaccarelli et al. 2023). However, research indicates that both climate change and land use changes jointly impact biodiversity and geographical distribution patterns (Song et al. 2021). As global warming progresses, climate change will not only alter surface temperatures and precipitation patterns but also significantly affect plant geographical distributions, causing plants to adjust their distribution in response to climate shifts. Additionally, habitat loss due to land use changes will exacerbate the impact of climate change on global species and ecosystems (Pant et al 2021). To adapt to climate change, plant communities in terrestrial ecosystems need to migrate to new suitable habitats that offer essential ecological comfort to maintain their lifecycle balance. Some plants have shown a trend of migrating to higher latitudes or elevations to track changes in their ecological niches (Burrows et al. 2014). Conversely, other studies have found that some plants are migrating to lower elevations and latitudes to adapt to changing environments (Lenoir et al. 2010). Climate change introduces high uncertainty in plant cultivation, and blind planting could impact quality and lead to resource allocation imbalances (He et al. 2021). While long-term field trials have traditionally been considered reliable for determining suitable planting areas, they are resource-intensive and require observation over multiple growth cycles (Li et al. 2019). Therefore, predicting potential suitable areas for plants under future climate change and understanding their cultivation potential in new environments is increasingly necessary.

Since the 1990s, human-induced declines in stratospheric ozone concentrations have been accompanied by an increase in ultraviolet (UV) levels compared to the past, which is likely to continue to increase in the future. This phenomenon poses a major potential threat to human health and global agricultural security (Tevini 2004). Plants are always exposed to UV-B radiation as they require light for photosynthesis. However, it is well known that exposure to UV-B radiation directly or indirectly triggers a variety of unfavorable responses that can inhibit plant growth and development (Yadav et al. 2020). Environmental factors and enhanced doses of UV-B radiation have effects on microorganisms, algae, plants, animals and human health (Farman et al. 1985). Studies have shown that UV-B radiation has a significant damaging effect on plants (Tevini and Teramura 1989). Plants, as stationary organisms, have developed a variety of morphological, physiological, biochemical and molecular level responses to various environmental changes, including increased UV radiation, over a long period of evolution. For example, plants adopt multiple protective strategies through specific UV-B signaling pathways, including increased leaf thickness, synthesis of UV-B-reflecting substances, elevated levels of antioxidants, and changes in UV-B uptake secondary metabolites at the cellular level (Yadav et al. 2020). By studying the future distribution range of plants under UV radiation, it is possible to gain a deeper understanding of the mechanisms of plant adaptation to UV-B and provide a theoretical basis for future plant variety improvement.

Rice (*Oryza sativa*) is one of the most important food crops globally, feeding more than half of the world’s population. It is the second most widely grown cereal crop after wheat (Carrijo et al. 2017; Fukagawa and Ziska 2019). China contributes nearly 30% of the world’s rice production, and approximately 90% of the world’s rice is produced in Asia, making China one of the largest rice producers globally (Samal et al. 2022). Rice plays a crucial role in human nutrition, as it is rich in minerals such as iron, phosphorus, and calcium, as well as vitamins B1 and B2, carbohydrates, and proteins. It is widely used in various dishes to meet human nutritional needs (Muratbek Kyzy et al. 2023). In addition to being a major food source, rice can also be used as a raw material for producing various products, including alcoholic beverages (Ito and Lacerda 2019; Meng et al. 2019) and sugar (Saithong and Lomthong 2019). Rice production is directly linked to economic stability and food security Zhu et al. (2024), having a profound impact on human survival and progress (Cao et al. 2018). However, rice growth is influenced by multiple factors, including genetic information, plant hormones, signaling molecules, nutritional status, and external environmental conditions such as light, temperature, and water (Ali and Baloch 2018; Mittler et al. 2004; Wang H. et al. 2023). Climate change, particularly extreme weather events like typhoons and high temperatures, poses a threat to rice supply (Lenaerts et al. 2019). Additionally, changes in soil nutrients, water quality, and temperature significantly affect rice growth and yield, with these factors collectively determining the growth and development performance of rice.

Species distribution models (SDMs) are essential predictive tools in conservation ecology, effectively studying the ecological relationships between species and their environments, They are capable of predicting species distributions under current and future climate scenarios (Hällfors et al. 2016). SDMs are crucial for assessing the impact of climate change on species distribution. By combining known species distribution data with environmental information, SDMs determine the ecological requirements of species and use statistical or machine learning methods to predict suitable areas under various spatial and temporal conditions. These models are widely used to understand the environmental needs of target species, simulating distribution ranges in multidimensional environments to predict potential suitable habitats under different environmental changes (Elith and Leathwick 2009). SDMs are applied in biodiversity conservation research, species invasion studies, evolutionary biology, and habitat suitability prediction (Barbet-Massin et al. 2018).

There are now dozens of SDMs, each with its own principles, algorithms, and predictive performance (Luo et al. 2017). Due to the different principles and algorithms used by each model, they each have their advantages and limitations. Additionally, the performance of each model can become unstable if the input data changes. Variations in data requirements and algorithms among different SDMs lead to differences in how environmental factors impact species distribution, introducing uncertainty into the simulation results (Guo et al. 2015). Ensemble models (EMs) improve the reliability of modeling results by combining the true data captured by individual models and minimizing their inherent errors (Dormann et al. 2018). In 2003, the integrated modeling platform Biomod, based on R software, was developed and has since been widely recognized and used (Bi et al. 2013). This package, available for free download from CRAN (cran.r-project.org), includes various species distribution modeling algorithms: GLMs (Guisan et al. 2002), generalized boosting models (GBMs) (Elith et al. 2008), generalized additive models (GAMs) (Guisan et al. 2002), classification tree analysis (CTA) (Vayssières et al. 2000), artificial neural networks (ANN) (Lek and Guégan 1999), surface range envelope (SRE) modeling (Busby 1991), flexible discriminant analysis (FDA) (Hastie et al. 1994), multivariate adaptive regression splines (MARS) (Friedman 1990), random forests (RF) (Breiman 2001) and MaxEnt (Lantschner et al. 2019). Biomod2 integrates these model results to provide more accurate species distribution predictions. Compared to single models, Biomod2 enhances prediction accuracy and increases the reliability of research findings by applying different statistical methods (Hao et al. 2019). Due to its ability to compensate for the limitations of individual models, Biomod2 has rapidly become a key tool for species distribution prediction in recent years (Zhao et al. 2021). Although each model has inherent flaws, weighting each model based on TSS (true skill statistic) or ROC (receiver operating characteristic curve) helps achieve optimal simulation performance in ensemble models (Shabani et al. 2016).

Research indicates that climate change and human activities are causing a gradual reduction in the suitable areas and habitats of most plants (Hao et al. 2019), threatening their reproductive capacity. This could also affect rice (*O. sativa*), potentially leading to food resource shortages. This study integrates the synergistic effects of climate change and land use changes to develop a comprehensive ecological niche model for *O. sativa*, identifying the main environmental factors limiting its distribution. The study also maps suitable areas for *O. sativa* and examines the impact of UV radiation on its suitable habitats. By considering climate and land use changes, the aim is to provide more effective references for the conservation and sustainable use of *O. sativa* resources and offer a framework for managing and planning other staple crops.

## 2 Material and methods

### 2.1 Species occurrence data

The distribution data for *O. sativa* was primarily gathered through resource-sharing platforms, including the Global Biodiversity Information Facility (GBIF, https://www.gbif.org/, accessed on April 5, 2024). Additionally, published literature and related reports were reviewed using keywords such as *O. sativa* in sources like Flora of China, provincial plant floras, and relevant checklists. A total of 9045 raw records were collected. To reduce the impact of spatial autocorrelation and sampling bias on the final predictions, the SDM toolbox (version 2.5) was used to process the sparse occurrence data, ensuring that only one distribution point was included per 1 km × 1 km grid cell (Zhang et al. 2024). This sparse point method was aligned with the resolution of environmental data to minimize the number of distribution points affected by spatial autocorrelation and to avoid model overfitting (Brown et al. 2017). After excluding records with uncertain information, ambiguous names, non-ground data, and duplicates, 140 valid distribution point coordinates were retained for further data processing and analysis. For ease of modeling, the distribution data file was converted to CSV format.

### 2.2 Environmental variables

Previous studies have not yet identified the primary environmental variables limiting the distribution of *O. sativa*. One objective of this research is to analyze how *O. sativa* adapts to environmental conditions. To achieve this, a broad selection of environmental variables was included, encompassing factors such as climate, soil, topography, and anthropogenic influences. A total of 24 environmental variables were chosen for habitat prediction. These included 19 bioclimatic variables (downloaded from the WorldClim database www.worldclim.org/data/worldclim21.html, accessed on April 18, 2024) and three topographic factors (elevation, aspect, and slope), obtained from the Geospatial Data Cloud (www.gscloud.org, accessed on April 1, 2024). The d1_ph_water data are from the Global Water Quality Database (GLOWAQ) (https://www.glowaq.org), the d1_usda data are from the United States Department of Agriculture (USDA) Soil Survey Geographic Database (SSURGO) (https://websoilsurvey.nrcs.usda.gov), hf_v2geo1 data from the Global Human Footprint Map (https://sedac.ciesin.columbia.edu), and uvb1_annual_mean_uv-b data from NASA’s OMI Satellite Observations Dataset (https://aura.gsfc.nasa.gov). The data were downloaded in raster format from the respective databases, standardized to the same grid size and coordinate system, and then converted to ASCII format using ArcGIS. Climate variables under the Representative Concentration Pathway (SSP2-4.5) scenario were used for future predictions. Future climate data were projected for three periods: the 2050s (average for 2041-2060), the 2070s (average for 2061-2080), and the 2090s (average for 2081-2100).

**Figure 1:**
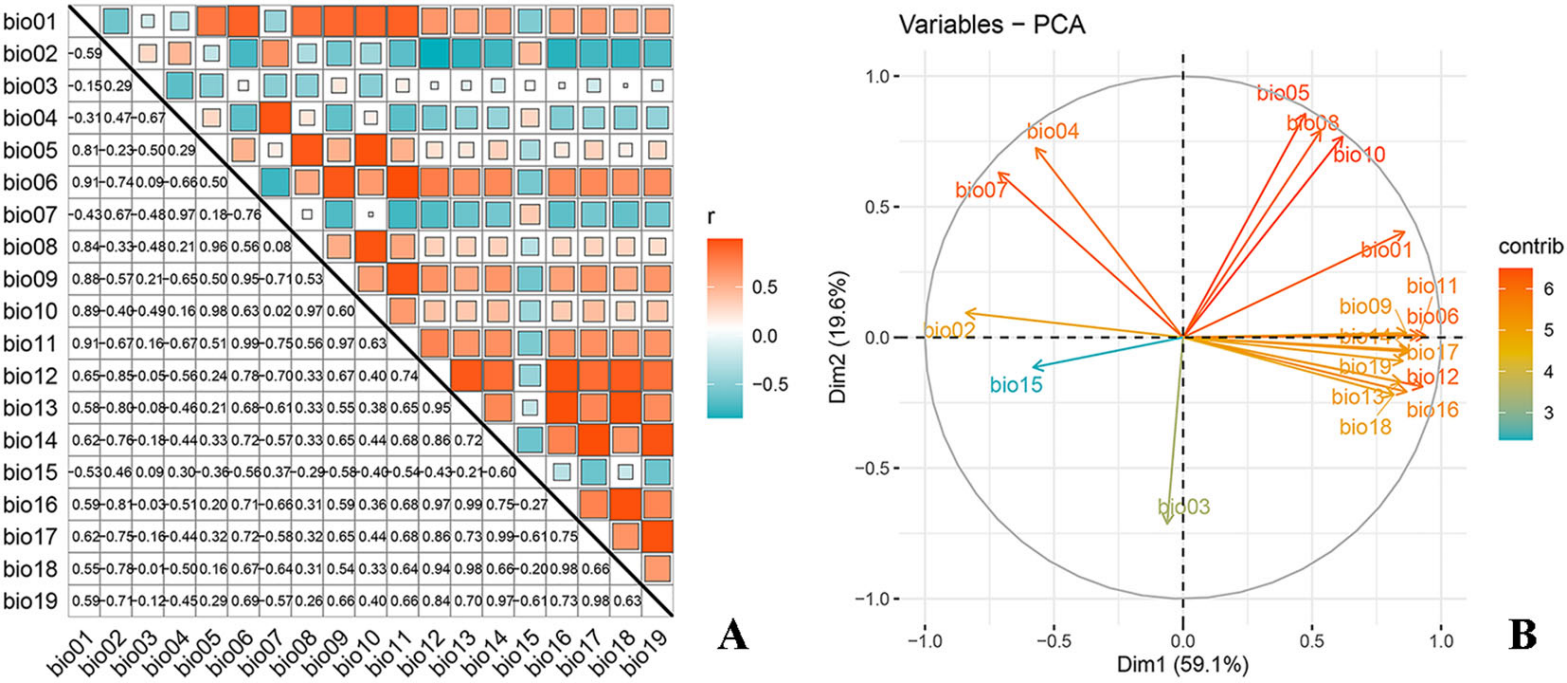
Correlation analysis **(A)** and PCA analysis **(B)** of environmental variables.

Correlation analysis and principal component analysis (PCA) were performed on 19 climatic factors using the R software (**Figure 1**) to address potential multicollinearity issues among environmental variables, which could lead to model overfitting and affect prediction accuracy. Variables with a correlation coefficient exceeding 0.8 were evaluated, and those with higher contributions to *O. sativa* distribution were prioritized for inclusion. Ultimately, the selected environmental variables included 8 bioclimatic indicators (bio01, bio03, bio04, bio05, bio06, bio09, bio11, and bio17), 1 topographic indicator (elevation), 2 soil indicators (d1_ph_water and d1_usda), and 1 anthropogenic indicator (hf_v2geo1) (**Table 1**). Additionally, the study separately modeled scenarios with and without UV radiation to analyze its effects.

**Table 1:** Environmental variables retained after screening.

Variable	Environment Variables	Unit
bio01	Annual mean temperature	°C
bio03	Isothermality ((bio02/bio07) × 100)	%
bio04	Temperature seasonality (standard deviation × 100)	–
bio05	Max temperature of warmest month	°C
bio06	Min temperature of coldest month	°C
bio09	Mean temperature of driest quarter	°C
bio11	Mean temperature of coldest quarter	°C
bio17	Precipitation of driest quarter	mm
elev	Elevation	m
d1_ph_water	Soil acidity	–
d1_usda	Soil USDA texture classification	–
hf_v2geo1	Human footprint and human activity impact index	–
uvb1_annual_mean_uv-b	Annual average ultraviolet radiation	W/m

### 2.3 Construction and evaluation of SDMs

Biomod2 is a comprehensive platform that establishes the relationship between species and environmental variables using statistical and machine learning algorithms. To meet Biomod2’s modeling requirements and better simulate actual distributions, 1000 pseudo-absence points were randomly selected outside the predicted suitable range for modeling (Cengic et al. 2020).

To assess the performance of the species distribution models, the distribution data for *O. sativa* was randomly divided into two subsets. One subset, containing 75% of the distribution data, was used for model training, while the remaining 25% served as a test dataset to evaluate model performance. To minimize errors from a single modeling approach, this process was repeated 10 times for each model, resulting in 100 individual modeling outcomes (Bi et al. 2022).

Model accuracy was evaluated using ROC curves and the True Skill Statistic (TSS). TSS, introduced by Allouche and Kadmon in 2006, is a widely recognized and effective evaluation metric for SDMs. It combines the advantages of Kappa statistics while addressing its limitations in species occurrence with unimodal response curves (Allouche et al. 2006). TSS values range from 0 to 1, with values greater than 0.7 indicating high predictive accuracy and values below 0.5 suggesting poor accuracy. ROC values range from 0 to 1, with 0 to 0.6 indicating poor predictive performance, 0.6 to 0.8 indicating moderate performance, 0.8 to 0.9 indicating good performance, and 0.9 to 1.0 indicating excellent performance. Models with ROC > 0.90 and TSS > 0.65 were selected. Finally, the EM model was used to determine the area of suitable habitat for *O. sativa* under current and future climate scenarios.

### 2.4 Data processing

The selected environmental and distribution data for *O. sativa* were input into the ensemble model, and the resulting data were visualized using ArcGIS 10.8. The zones were classified based on the suitability index P, normalized by ArcGIS. A threshold of P ≥ 0.05 was used to indicate suitable habitats where the species can survive. Based on this threshold, the suitability was categorized into four classes: unsuitable (0-0.1), low suitability (0.1-0.3), moderate suitability (0.3-0.6), and high suitability (0.6-1). Grid calculations were performed to tabulate the area of each reclassified suitability category, and the weights for each zone were determined. Subsequently, the area of each layer was adjusted according to the actual land area cutout. Using the SDM_Toolbox_v2.5, the loss (areas currently suitable but becoming unsuitable in the future), stable (areas suitable now and in the future), and gain (areas currently unsuitable but becoming suitable in the future) regions for *O. sativa* were identified for different time periods.

## 3 Results

### 3.1 Evaluation of model prediction accuracy

**Figure 2:**
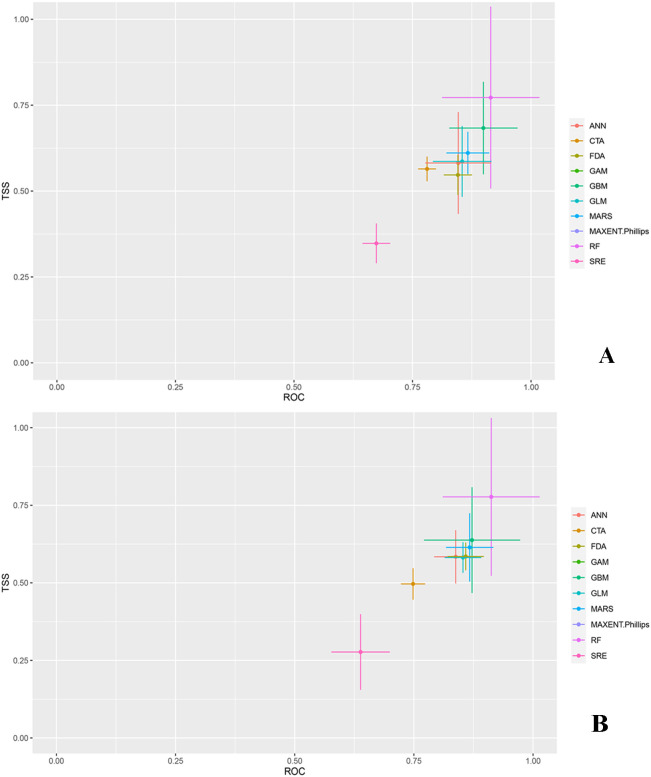
True skill statistics (TSS) and receiver operating characteristic curve (ROC) of 10 SDMs. **(A)** denotes the TSS and receiver operating characteristic (ROC) values of the model without UV variables, and **(B)** denotes the TSS and receiver operating characteristic (ROC) values of the model with UV variables. ANN (Artificial Neural Network), CTA (Classification Tree Analysis), FDA (Flexible Discriminant Analysis), GAM (Generalized Additive Model), GBM (Gradient Boosting Machine), GLM (Generalized Linear Model), MARS (Multivariate Adaptive Regression Splines), MAXENT (Maximum Entropy), RF (Random Forest), SRE (Species Distribution Modelling using Rule-based Ensembles).

To rigorously assess the accuracy of the models in predicting the distribution of *O. sativa*, we used data on the distribution of *O. sativa* in China, combined with environmental variables, and ran the models 10 times to obtain TSS and receiver operating characteristic (ROC) values for the 10 models. The prediction accuracy of individual models was assessed based on ROC and TSS values (**Figure 2**). The results from the ensemble species distribution models revealed variations in prediction accuracy among the 10 models. **Figure 2** indicates that GAM and MAXENT had the poorest prediction accuracy, failing to accurately simulate suitable areas. In contrast, RF had the highest average TSS and ROC values, demonstrating the best accuracy and stability.

Models with ROC values > 0.9 and TSS values > 0.65 were selected. For the no UV variable scenario, RF, GBM, ANN, and MARS were chosen, resulting in an ensemble model (EM) with ROC = 0.976 and TSS = 0.825 (**Table 2**). For the UV variable scenario, RF, GBM, and MARS were selected, leading to an EM with ROC = 0.977 and TSS = 0.836 (**Table 2**). Except for RF, the ROC and TSS values of the ensemble models were higher than those of individual models, indicating superior prediction accuracy for *O. sativa* suitable areas. Given its excellent performance, we chose the EM model for further visualization and analysis.

**Table 2:** The mean value of receiver operating characteristic curve (ROC) and true skill statistic (TSS) of different model algorithms.

Model Name	Model Code	ROC		TSS	
		no ultraviolet radiation	ultraviolet radiation	no ultraviolet radiation	ultraviolet radiation
Random forest model	RF	0.9995	1	0.9955	0.9970
Generalized boosting model	GBM	0.9575	0.9545	0.7890	0.7810
Artificial neural network model	ANN	0.9040	0.8730	0.7075	0.6355
Multivariate adaptive regression spline model	MARS	0.9025	0.9000	0.6540	0.6650
Generalized linear model	GLM	0.8850	0.8835	0.6250	0.6115
Flexible discriminant analysis model	FDA	0.8705	0.8760	0.5965	0.6025
Classification tree analysis model	CTA	0.7725	0.7675	0.5450	0.5350
Surface range envelope model	SRE	0.6940	0.6905	0.3880	0.3810
Generalized additive model	GAM	NA	NA	NA	NA
Maximum entropy model	MaxEnt	NA	NA	NA	NA
Ensemble model	EM	0.9760	0.9770	0.8250	0.8360

### 3.2 Evaluation of environmental variables

The weights of the nine environmental variables varied (**Table 3**). In the absence of UV variables, bio01, bio04, bio06, hf_v2geo1, bio11 and altitude were the most important environmental factors affecting *O. sativa*. In the presence of UV variables, bio01, bio04, bio06, bio11, hf_v2geo1 and uvb1_annual_mean_uv.b were the most important environmental factors affecting *O. sativa*. Regardless of the UV variables, the biodiversity indices bio01, bio04 and bio06 had the highest weights and were the most important environmental factors affecting *O. sativa*. Overall, temperature, UV intensity and elevation play a crucial role in determining the habitat range of *O. sativa*.

**Table 3:** Importance weight of environmental variables.

Variable	Importance weight	
	no ultraviolet radiation	ultraviolet radiation
bio01	0.3971	0.3874
bio03	0.0844	0.0962
bio04	0.3577	0.3492
bio05	0.1166	0.1153
bio06	0.3050	0.2922
bio09	0.1111	0.1398
bio11	0.1783	0.2295
bio17	0.1385	0.1474
d1_ph_water	0.0294	0.0325
d1_usda	0.0155	0.0236
elev	0.1655	0.1572
hf_v2geo1	0.2472	0.2151
uvb1_annual_mean_uv-b	/	0.1708

Based on these results, RF shows better performance compared to other models. We used the response curves generated by RF to explore the relationship between current environmental factors and the probability of occurrence. **Supplementary Figure S1** illustrates that the habitat suitability of *O. sativa* exhibits a nonlinear relationship with various environmental variables. Whether or not the UV variable is included, habitat suitability for *O. sativa* improves as the annual mean temperature (bio01) exceeds 15°C. Similarly, habitat suitability increases as the lowest temperature of the coldest month (bio06) rises above 10°C. The suitability of *O. sativa* for different habitats first decreases and then increases with seasonal temperature changes, eventually stabilizing. The response curves for d1_ph_water, bio03, and d1_usda show that these variables have relatively stable probabilities and thus have a minor impact on the suitable distribution of *O. sativa*. The presence or absence of UV variables significantly affects the impact of the precipitation during the driest season (bio17) on the suitable distribution of *O. sativa*. Higher values of uvb1_annual_mean_uv.b lead to a decrease in habitat suitability for *O. sativa*.

### 3.3 Geographical distribution of *O. sativa* under current and SSP2-4.5 climates

**Figure 3:**
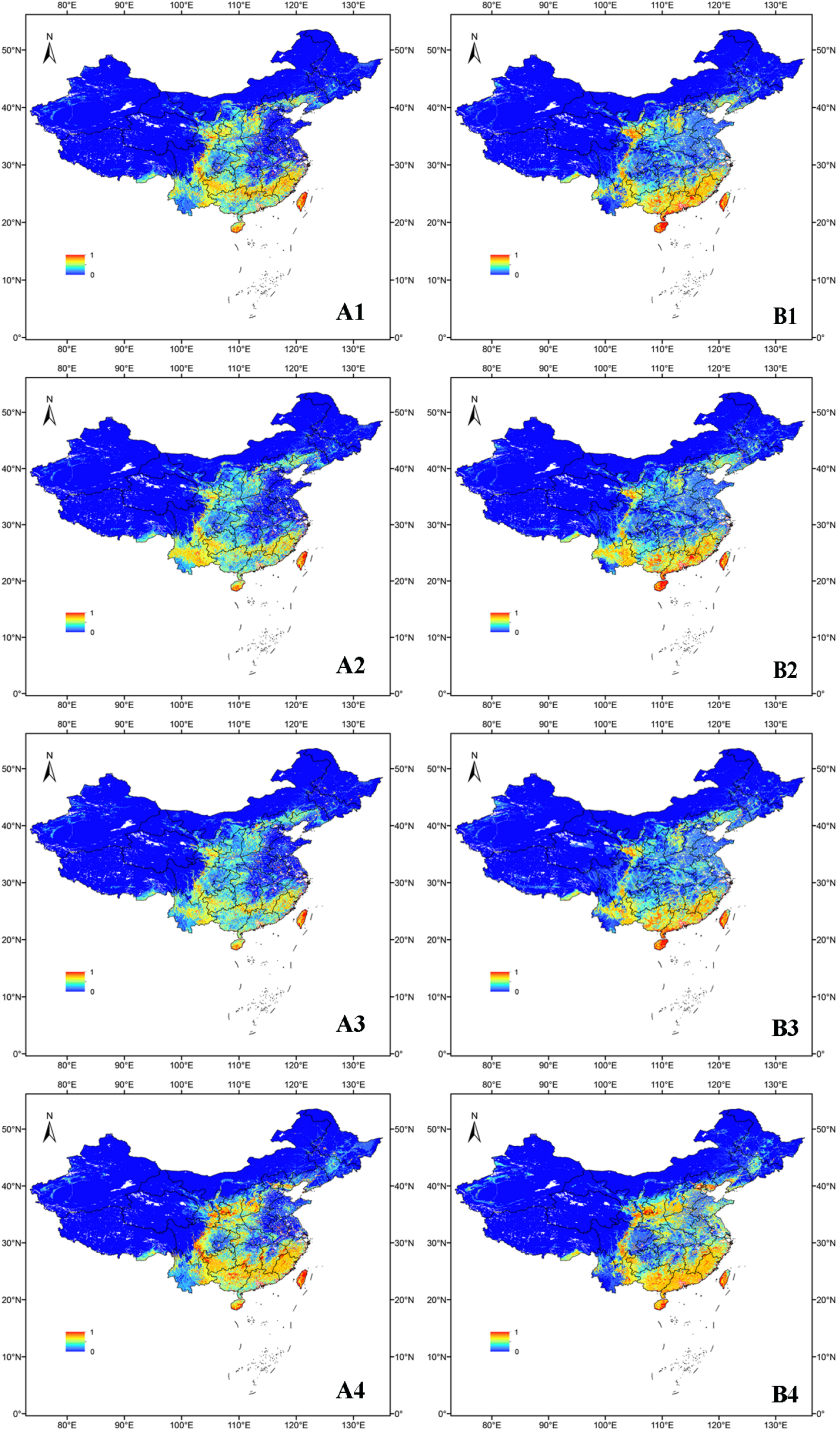
Suitability distribution of *O. sativa* in the scenarios SSP2-4.5 and current. **(A)** denotes the distribution without the UV variable and **(B)** denotes the distribution with the UV variable.1 denotes the distribution in the 2050s under SSP2-4.5, 2 denotes the distribution in the 2070s under SSP2-4.5, 3 denotes the distribution in the 2090s under SSP2-4.5, and 4 denotes the distribution under the current scenario.

**Figure 3** shows that under current climate conditions, the suitable regions for *O. sativa* are primarily located in the southern part of China. With the inclusion of UV variables, high suitability regions in the current climate have decreased in the northeastern part of Taiwan, as well as in Fujian, Jiangxi, Hunan, Guangxi, Guangdong, Shanxi, Yunnan, Guizhou, and Sichuan. In contrast, high suitability regions have increased in Gansu, Hebei, and Hainan. The medium and low suitability areas in Yunnan, Guizhou, Sichuan, Chongqing, Hunan, and Hubei have decreased, while regions like Shandong, Henan, Hebei, Jiangsu, and Anhui have seen increases.

In the 2050s SSP2-4.5 scenario, the highly suitable areas for O. sativa with UV variables were mainly located in southeastern China. The area of highly suitable areas was larger than that of the scenario without the UV variable, while the area of unsuitable areas was slightly larger in eastern and central China. By the 1970s, under the SSP2-4.5 scenario, the moderately suitable areas of *O. sativa* gradually decreased and the highly suitable areas slightly increased.

### 3.4 Changes of *O. sativa* distribution under future climatic conditions

Compared to the current climate conditions, the total suitable habitat area for *O. sativa* shows a decreasing trend under the SSP2-4.5 scenario (**Table 4**). The contraction of suitable habitats is more significant without UV variables compared to with UV variables, and the expansion of suitable habitats is less under no UV variables, with a notable reduction by half (47,764.8866 km²) by the 2070s. The “No occupancy” areas remain almost unchanged across the three time periods, while the “No change” areas decrease with time, being larger without UV variables compared to with UV variables.

**Table 4:** Distribution change of *O. sativa* distribution in 2050s, 2070s and 2090s under SSP2-4.5.

SSP2-4.5	Area (km^2^)							
	Range expansion		No occupancy		No change		Range contraction	
	No ultraviolet radiation	ultraviolet radiation	No ultraviolet radiation	ultraviolet radiation	No ultraviolet radiation	ultraviolet radiation	No ultraviolet radiation	ultraviolet radiation
2050s	83980.8050	118550.5452	8780971.2388	8800288.1100	162174.2651	148053.1435	154303.4760	114537.9861
2070s	47764.8866	105689.7788	8817187.1572	8813148.8765	138407.5687	130871.1595	178070.1724	131719.9701
2090s	43958.0998	98384.8634	8820993.9440	8820453.7918	134163.5157	129044.9307	182314.2254	133546.1989

**Figure 4:**
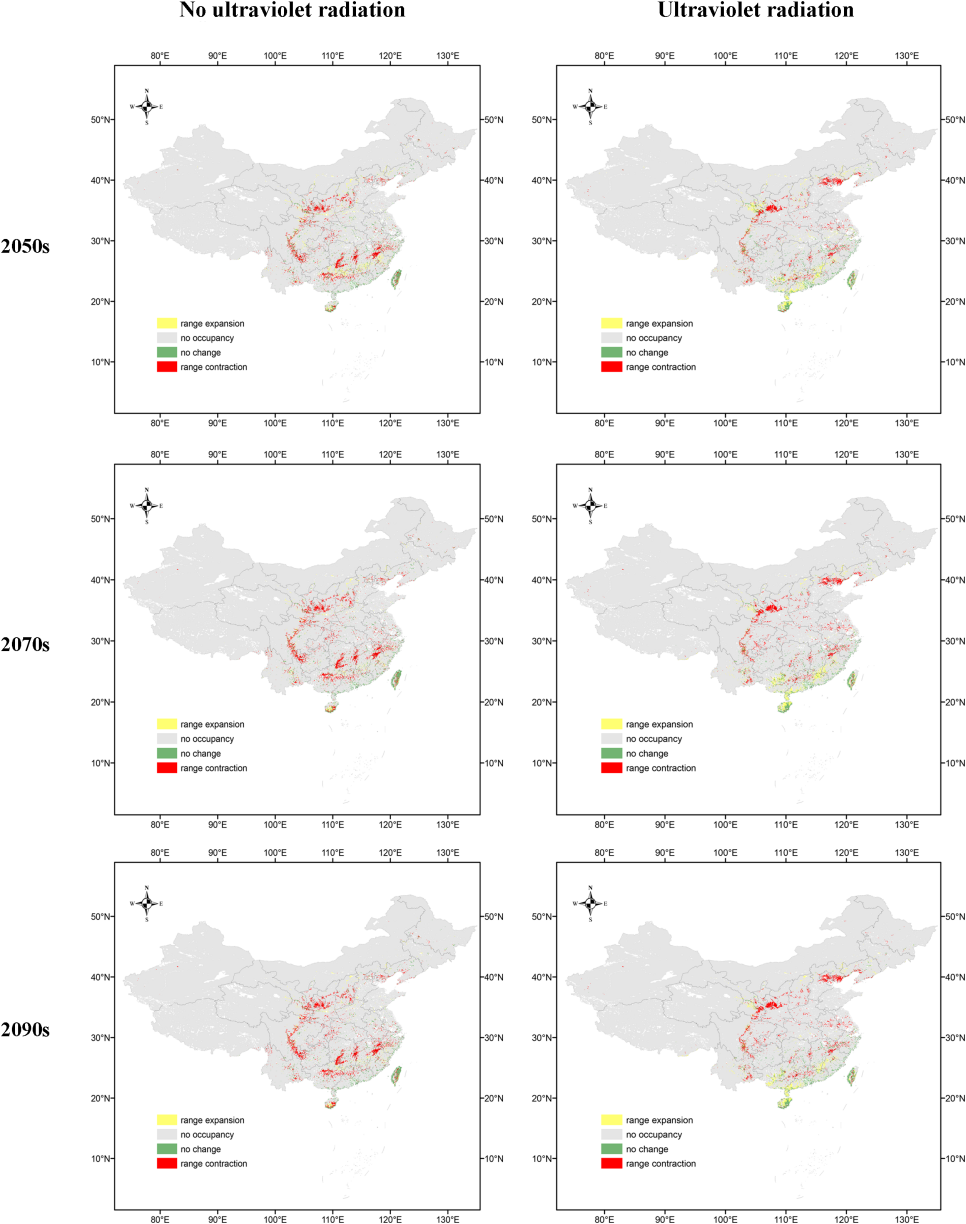
Distribution change of *O. sativa* in the scenarios SSP2-4.5.

**Figure 4** illustrates that in the 2050s, the suitable habitat expansion range for *O. sativa* is greater with UV variables than without, particularly in Hainan, Shaanxi, and Gansu. The contraction of suitable habitats with UV variables is concentrated in Shaanxi, Shanxi, Hebei, and Beijing, whereas, without UV variables, there is additional contraction in Hunan, Jiangxi, Zhejiang, and Fujian. The “No change” areas are more extensive without UV variables than with UV variables. In the 2070s and 2090s, the expansion range of suitable habitats for *O. sativa* continues to decrease in both scenarios, and the contraction range continues to increase. The “No change” areas remain more extensive without UV variables compared to with UV variables.

Overall, while the suitable habitat range for *O. sativa* is expected to decrease in the future, the presence of UV variables results in a greater range of suitable habitats compared to scenarios without UV variables. This indicates that UV radiation is an important environmental factor influencing the distribution of *O. sativa*.

## 4 Discussion

Predictions of suitable habitat distribution are influenced by a variety of factors, including the availability of species distribution data, the choice of species distribution models (SDMs), and the type of environmental variable. Models that combine physically present and absent data generally perform better than models that rely only on physically present data when predicting the distribution of suitable habitat (Brotons et al. 2004). In addition to subtle differences in distribution records, differences in the choice of environmental variables, variable screening methods, and parameter settings can affect the bias in the final prediction results. Too many distribution points may lead to model overfitting, while too similar climate data may lead to large covariances in model results (Abdulwahab et al. 2022; Araújo and Peterson 2012). It is important to select the least correlated and biologically plausible predictors for prediction (Weng et al. 2020). The study screened 13 environmental factors for prediction through correlation analysis and principal component analysis (PCA). In order to ensure the accuracy of the prediction of *O. sativa*’s suitable land distribution, the data collection method was used in the study. Differences in the algorithms of different SDMs, as well as differences in the effects of various environmental factors on the distribution of species, led to some uncertainty in the prediction results of each SDM. Even when using the same dataset, different single models may produce different predictions. A potentially effective approach is to use a hybrid model, such as Biomod2, which combines a set of algorithms that use the same initial dataset and parameterization to make predictions (Di Febbraro et al. 2023; Thuiller 2014). In this study, four different SDMs (RF, GBM, ANN, and MARS) were integrated to improve the accuracy of the integrated model and overcome the limitations of the single-model approach to obtain a more accurate characterization of the geographic distribution of *O. sativa*. In this study, the accuracy of the models was evaluated using both ROC and TSS methods, and higher ROC values and less variability were obtained using the ensemble model compared to using individual models, which confirms the advantage of using the ensemble model to predict species occurrence (Araújo and New 2007; Breiner et al. 2015; Marmion et al. 2009). This study mapped the potential distribution of *O. sativa* under current and future climatic conditions. The TSS values of both the combined and RF models were above 0.80, representing excellent predictive ability (Koo et al. 2017).

Climate, topography, and soil play a key role in plant survival. Suitable temperature and moisture are essential for plants to carry out their basic physiological activities. At the same time, topographical factors such as slope, aspect, and elevation indirectly affect plant growth by influencing localized temperature, hydrological conditions, and soil properties. The results of this study showed that environmental variables such as bio01, bio04, bio06, hf_v2geo1, bio11, uvb1_annual_mean_uv.b, and elev had a greater effect on the distribution of *O. sativa*. *O. sativa* is a thermophilic plant, but too much elevated temperatures can lead to a reduction in the yield of *O. sativa*. There were differences in the rate of reduction, and higher temperatures can also lead to a decrease in the quality of rice. The environmental variables bio01 and bio17 have large weights, and they have a significant impact on the amount of water and heat required for *O. sativa* to expand northward. *O. sativa* is primarily adapted to grow in warm and humid environments, while dry and cold winters act as a limiting factor in its expansion range. The relatively wide range of bio04 values suggests that *O. sativa* may be able to adapt to regions with large temperature fluctuations. Larger isotherms imply that *O. sativa* is able to enhance photosynthetic efficiency at high daytime temperatures, while lower nighttime temperatures help to reduce energy consumption for respiration, thus promoting metabolite accumulation and plant growth (Li et al. 2008). Precipitation has a significant effect on above and below ground organs of plants. studies have shown that insufficient water may lead to reduced plant biomass (Wan et al. 2022), prolonged germination (Thabet et al. 2018), reduced leaf mass (Wan et al. 2022) and reduced root density (Funk et al. 2021), In contrast, adequate water helps to increase root length and leaf area (Song et al. 2022), and promotes plant germination (Plazek et al. 2018). In addition, altitude has an effect on the accumulation of metabolites and nutrients in below- and aboveground organs, which usually interacts with factors such as nutrient availability, water content (Zuo et al. 2022), temperature, UV intensity, CO_2_ concentration, and UV-B intensity (Zhang et al. 2018).

Solar radiation is the main influence on the growth of most plants (Jiang et al., 2020; Zuo et al., 2022). Solar radiation is one of the driving forces of photosynthesis, through which vegetation converts solar energy into chemical energy to synthesize organic matter needed for plant growth (Xu et al. 2024). Elevated UV intensity leads to decreased soluble protein content in *O. sativa* leaves, decreased photosynthetic activity, growth inhibition, shorter plants, and decreased yield (Xiaohua 1994). The study showed that the extent of the effect of UV on yield and yield components was greatly influenced by environmental factors such as temperature and sunshine, and that the inhibitory effects of UV on plant height, stem number, and number of spikes varied from year to year, with the inhibitory effects of UV-B being weakened in high-temperature, multi-daylight years, and becoming stronger in low-temperature, low-light years. The results of the study on the effect of UV on the quality of *O. sativa* indicated that UV exposure during the reproductive growth period and after tasseling had a significant effect on the size of rice grains, resulting in a significant reduction in the proportion of large rice grains (Wen-Hui et al. 2003). *O. sativa* leaves exposed to higher UV radiation were shorter and thicker, the dry weight of the plant body, i.e. biomass, was reduced and its cells and tissues were damaged. Total intake of root activity, protein and nutrients was reduced and stomata opened less and often closed (Jianping 1992). Enhanced UV radiation can also cause shorter plants, delayed fertility, reduced tiller number, decreased biomass and yield (Dai et al. 1992; Teramura et al. 1991), and smaller grain size of UV-irradiated rice (Kumagai et al. 2001). This is consistent with the study in this paper that future UV enhancement will reduce the suitable distribution range of *O. sativa*. However, we found in the effects of rice with and without UV that the contraction of the suitable habitat range was greater in the case of the no UV variable than in the case of the UV variable, and the expansion of the suitable habitat range was less in the case of the no UV variable than in the case of the UV variable, These results suggest that there may exist some kind of UV protective mechanism in rice plant to tolerate UV-B exposure (Roy and Roy 2013). Some substances in rice regulate flavonoid accumulation and are involved in UV-B tolerance in rice (Zhang et al. 2022), UV-B radiation induces the synthesis of rice proanthocyanidins B2 and C1 (Li et al. 2023), The enhanced UV-B radiation contributed to suppressing the M. oryzae infection and alleviating its damage to the photosynthesis of rice leaves (Zhang et al. 2024). Possible mechanisms of plant acclimatization to UV-B include changes in their structure, such as anatomical changes in the epidermal layer and epidermal waxes, increased leaf thickness and weight, and increased absorption of UV-B pigments (Bornman et al. 2015).

In this study, the effects of UV radiation on rice were explored in depth for the first time using a combination of models. The effects of UV on the future growth of rice were assessed by combining different modeling approaches. This innovative research approach not only provides more accurate prediction results, but also provides important theoretical support for future strategies to cope with rice cultivation and variety improvement in the context of UV enhancement.

Studies have shown that *O. sativa* exists in a wide range of high suitability habitats in Hainan, Taiwan and other provinces and regions, suggesting that the species has a high demand for hydrothermal environments. However, due to the complexity of environmental factors affecting plants, it is difficult to comprehensively access all environmental variables affecting the distribution of *O. sativa* and precisely define their suitability zones. In addition, the application of these predictions to actual planting and restoration requires consideration of factors in land use and its surroundings, such as water quality, vegetation cover, and human activities. Changes in land use, especially due to land use for economic purposes, have also led to a reduction in suitable habitat (Zhang et al., 2018). Reduction in forest cover and fragmentation of highly forested protected areas due to changes in ecological distribution may result in the loss of significant amounts of currently suitable habitat (Jia et al. 2020). In future scenarios, the suitable area for *O. sativa* will decline significantly. In the future, global temperatures are expected to continue to rise, extreme weather events will be frequent, and human activities will intensify (Wang X. et al. 2023). Therefore, it is expected that the suitable distribution of *O. sativa* will continue to shrink. Our results are consistent with those of *Lonicera oblata* (Wu et al. 2021).

## 5 Conclusions

In this study, we assessed the distribution of *O. sativa* in China. Potential distribution areas of *O. sativa* under current and future climatic conditions with and without the influence of UV variables were predicted. An ensemble model (EM) for predicting suitable habitats for *O. sativa* under current and future climate scenarios (SSP2-4.5) was developed by modeling and predicting four sdms, RF, GBM, ANN and MARS, selected under no UV variables, and three sdms, RF, GBM and MARS, selected under UV variables. Compared with individual models, the ROC of EM was 0.976 and 0.977, and the TSS was 0.825 and 0.836, respectively, and its prediction was more accurate. The results showed that the main environmental factors affecting its distribution were bio01, bio04, bio06, hf_v2geo1, bio11, and uvb1_annual_mean_uv.b. Under the current climatic conditions, the habitats of *O. sativa* are very suitable mainly distributed south of the Yangtze River. Under future climate scenarios, which will reduce the potential distribution of *O. sativa*, the contraction range of suitable habitat under the no UV variable is greater than in the case with the UV variable, and the expansion range of suitable habitat under the no UV variable is less than in the case with the UV variable. The prediction was highly reliable and accurately predicted the suitable range and extent of *O. sativa*, which is important for the future conservation of *O. sativa*. This study is of great significance for the conservation, management, introduction and cultivation of *O. sativa*, and it can also provide a reference for the study of other food crops in China.

## Data Availability

The datasets presented in this study can be found in online repositories. The names of the repository/repositories and accession number(s) can be found in the article/**Supplementary Material.**
