# Supplementary figures and images for: Effects of ultraviolet radiation as a climate variable on the geographic distribution of Oryza sativa under climate change based on Biomod2
Source: Front Plant Sci. 2025 Apr 16;16:1552770. doi: 10.3389/fpls.2025.1552770 (PMC12041074; doi:10.3389/fpls.2025.1552770)

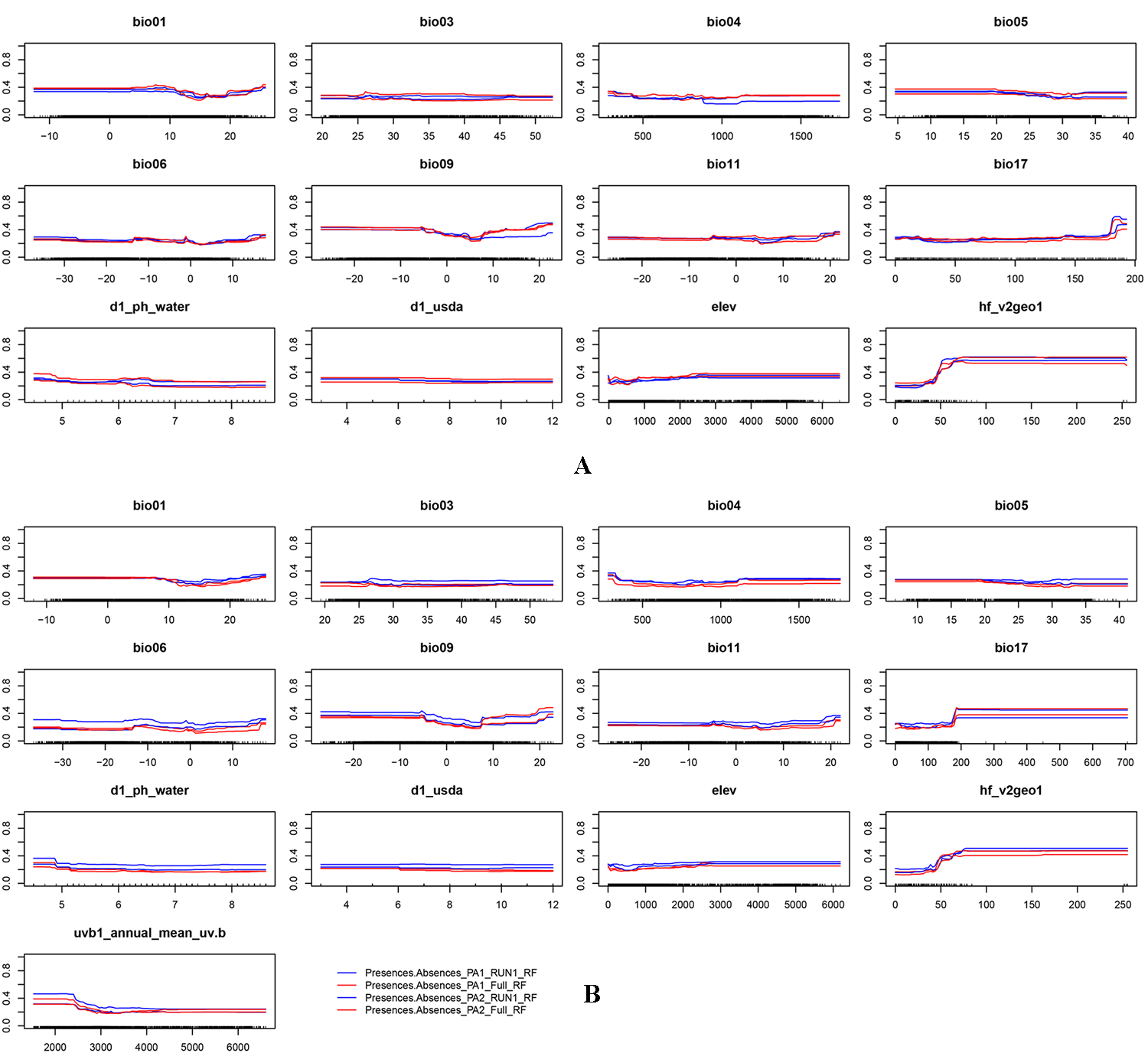

Supplement: Supplementary file 1 [file Image1.tif]
